# In Vivo Remodeling of Altered Autophagy-Lysosomal Pathway by a Phosphopeptide in Lupus

**DOI:** 10.3390/cells9102328

**Published:** 2020-10-20

**Authors:** Fengjuan Wang, Inmaculada Tasset, Ana Maria Cuervo, Sylviane Muller

**Affiliations:** 1 Biotechnology and Cell Signaling, CNRS-University of Strasbourg, Illkirch, France/Laboratory of Excellence Medalis, Institut de Science et d’Ingénierie Supramoléculaire, 67000 Strasbourg, France; bethy.fengjuan@gmail.com; 2 Department of Developmental and Molecular Biology, Albert Einstein College of Medicine, Bronx, NY 10461, USA; inmaculada.tasset@einsteinmed.org (I.T.); ana-maria.cuervo@einsteinmed.org (A.M.C.); 3 Institute for Aging Studies, Albert Einstein College of Medicine, Bronx, NY 10461, USA; 4 Fédération Hospitalo-Universitaire (FHU) OMICARE, Fédération de Médecine Translationnelle de Strasbourg (FMTS), Strasbourg University, 67000 Strasbourg, France; 5 University of Strasbourg Institute for Advanced Study (USIAS), 67000 Strasbourg, France

**Keywords:** chaperone-mediated autophagy, lysosomes, lupus, MRL/lpr mice, P140 peptide

## Abstract

The phosphopeptide P140/Lupuzor, which improves the course of lupus disease in mice and patients, targets chaperone-mediated autophagy (CMA), a selective form of autophagy that is abnormally upregulated in lupus-prone MRL/lpr mice. Administered intravenously to diseased mice, P140 reduces the expression level of two major protein players of CMA, LAMP2A and HSPA8, and inhibits CMA in vitro in a cell line that stably expresses a CMA reporter. Here, we aimed to demonstrate that P140 also affects CMA in vivo and to unravel the precise cellular mechanism of how P140 interacts with the CMA process. MRL/lpr mice and CBA/J mice used as control received P140 or control peptides intravenously. Lysosome-enriched fractions of spleen or liver were prepared to examine lysosomal function. Highly purified lysosomes were further isolated and left to incubate with the CMA substrate to study at which cellular step P140 interacts with the CMA process. The data show that P140 effectively regulates CMA in vivo in MRL/lpr mice at the step of substrate lysosomal uptake and restores some alterations of defective lysosomes. For the first time, it is demonstrated that by occluding the intralysosome uptake of CMA substrates, a therapeutic molecule can attenuate excessive CMA activity in a pathological pro-inflammatory context and protect against hyperinflammation. This recovery effect of P140 on hyperactivated CMA is not only important for lupus therapy but potentially also for treating other (auto)inflammatory diseases, including neurologic and metabolic disorders, where CMA modulation would be highly beneficial.

## 1. Introduction

It has long been recognized that lysosomes are at the heart of inflammation. In systemic lupus erythematosus (SLE), for instance, some defects have been described dealing with the composition and intrinsic activity of lysosomes that may affect the many functions of these central intracellular organelles [1,2,3,4,5]. In line with these observations, some autophagy alterations, mostly of macroautophagy, were demonstrated in the lymphocytes of two lupus-prone murine models of different major histocompatibility complex (MHC) haplotypes, namely Murphy Roths large/lymphoproliferation (hereafter named MRL/lpr) and (NZBxNZW)F1, as well as in the T and B lymphocytes of patients with lupus [6,7,8,9]. Macroautophagy deregulation was found to occur in several organs and tissues, notably in the spleen, lymph nodes, salivary glands and peripheral blood. While apparently dispensable for B-cell development, it was claimed that macroautophagy plays a major role in plasma cell survival, B-cell receptor trafficking and antigen presentation, and long-term autoantibody secretion [10,11]. Macroautophagy is also centrally involved in the maintenance and survival of memory CD4 and CD8 T cells with foreseeable consequences on the autoimmune response [12]. The underlying reasons for macroautophagy dysfunctions in lupus are not known but several independent investigations have identified risk loci spanning autophagy-linked genes in lupus patients [13,14,15].

Some defects have also been described in lupus regarding chaperone-mediated autophagy (CMA), a selective autophagic pathway mediated by the chaperones HSPA8/HSC70 and HSP90AA1, and by the lysosome-associated membrane protein 2A (LAMP2A). LAMP2A acts as a receptor at the lysosome and allows unfolded proteins to be delivered into the lysosomal lumen via a LAMP2A multimeric translocation complex. Only subsets of cellular lysosomes, those that contain HSPA8 in their lumen, are competent for CMA activity [16,17,18]. Together with LAMP2A, lysosomal HSPA8, which is also implicated in endosomal microphagy [19], represents a rate-limiting protein in CMA and this chaperone is crucial for the selective degradation of cytosolic proteins in lysosomes [17,19,20]. Model mice with compromised CMA in T cells display altered T-cell responses [21].

The existing results linking CMA and lupus were essentially provided by investigations using MRL/lpr lupus-prone mice [22] and there are no available data in patients with lupus. In these mice spleens, the expression of several lysosomal proteins (LAMP2A, LAMP1, cathepsin D) was found to be increased in purified B cells [22], and the levels of HSPA8 at the surface of several MRL/lpr cell subsets, notably activated lymphocytes, were abnormally raised [23,24]. In the salivary glands of these mice, however, the expression of LAMP2A, and of several macroautophagy-related proteins, namely membrane-associated microtubule-associated protein light chain 3-II (LC3-II/MAP1LC3-II), sequestosome 1 (SQSTM1)/p62 and autophagy-related (ATG) protein 12 was significantly diminished [9]. Quantitative gene expression analyses in MRL/lpr mice revealed differences in the transcription of hspa8 and lamp2 genes according to cell subsets and tissues [22,24]. Hspa8 mRNA expression was found to be raised in splenocytes (but not in lymph node cells and thymocytes) while lamp2a mRNA expression, the only spliced variant of the lamp2 gene required for CMA, remained unchanged or marginally enhanced in B cells from MRL/lpr mice compared to healthy CBA/J mice. MRL/lpr lysosomes exhibit some intrinsic defects, both in the spleen and salivary glands. They notably display a significantly higher luminal pH on average [3,9,22] with predictable consequences regarding the reduced activity of lysosomal hydrolases (e.g., cathepsins).

Some years ago, we discovered a peptide called P140, which exhibits protective activity in MRL/lpr mice and patients with SLE [25,26]. This synthetic phosphopeptide, issued from the cognate sequence 131–151 of the U1-70K spliceosomal protein, is currently being evaluated in phase III-clinical trials for lupus. One of our seminal findings was the discovery that P140 binds HSPA8 [23]. It interacts with the HSPA8 ATPase domain (*N*-terminal nucleotide-binding domain) leading to in vitro alterations of the HSPA8-HSP90AA1-bearing heterocomplex integrity, a loss of HSPA8 folding functions by a mechanism that likely involves HSPA8 ATPase activity and in cellulo affects the vital nuclear translocation of HSPA8 and associated client proteins in heat-shocked cells [22,23,27].

P140 enters B cells by the endolysosomal clathrin pathway and homes in on lysosomes [22,28]. Following an intravenous administration of P140 into MRL/lpr mice, the expression of several autophagy and lysosomal markers that is deregulated in the spleen and salivary glands (MAP1LC3-II, SQSTM1, LAMP2A, HSPA8 and cathepsin D) returns to the basal level measured in non-autoimmune mice [9,22,24]. In parallel, the density of MHC-II molecules that are raised at the surface of MRL/lpr B cells is rescued by P140 administration [24]. The same effect of P140 on MHC-II molecule expression was also observed using peripheral blood mononuclear cells from SLE patients [28]. It is assumed that by lowering the number of excessive MHC-peptide complexes to autoreactive T cells, the latter will lose their capacity to signal B cells with the consequence of diminishing the number/activity of auto-antibody-secreting plasma cells, hence the production of deleterious autoantibodies. The weaker activity of autoreactive CD4 T cells, the lower number of plasma cells and the drop of autoantibody reactivity have all been demonstrated in mice and patients with lupus [9,15,25,26,28,29].

Besides its direct effect on HSPA8 shown in vitro, P140 provokes a downregulation of macroautophagic flux that is hyperactivated in MRL/lpr splenic B cells [24]. The direct effect of P140 on CMA was measured in vitro using fibroblast cell lines that stably express photoswitchable CMA reporters. Using these original in vitro assays [30,31], P140 but not P140 analogues such as its scrambled form (ScP140) and others, were shown to significantly decrease CMA activity [3,22].

Even though we know that P140/Lupuzor exerts efficient therapeutic effects in mice and patients with lupus, virtually nothing is known about its in vivo protecting effects on lysosomes and influence on CMA. To address these central questions and fill these mechanistic gaps, we conducted a set of experiments in which we measured the extent of lysosomal and CMA defects and their possible remediation after an intravenous administration of P140 peptide into MRL/lpr mice. First, we focused our studies on the lysosomal defects and showed that some of them are relieved by treating mice with P140. Then, using purified lysosomes from P140-treated MRL/lpr mice, we directly analyzed their CMA activity, and confirmed for the first time the capacity of P140 to affect CMA in vivo. These results are pivotal since we are currently using P140 peptide not only in the setting of lupus but also in other autoinflammatory diseases where CMA modulation could be highly beneficial [9,32,33]. P140 peptide is the only pharmacological tool that selectively targets and decelerates CMA hyperactivity.

## 2. Materials and Methods

**Figure 1:**
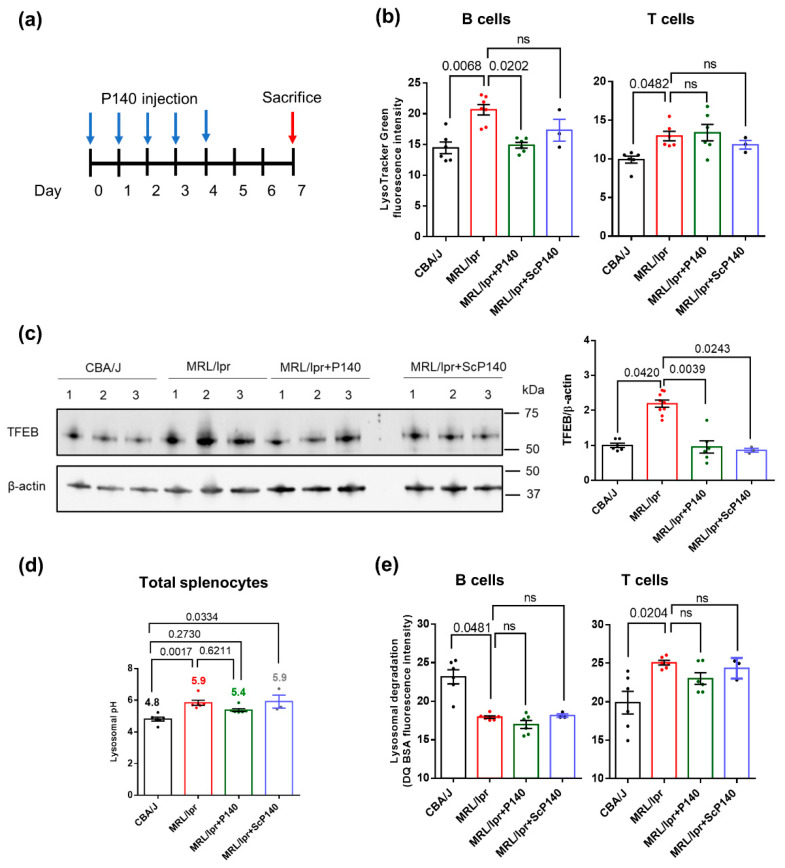
Lysosomal dysfunction in lupus: Total splenocytes were isolated from CBA/J (*n* = 6), MRL/lpr mice (*n* = 6–9) and MRL/lpr mice that received P140 (*n* = 6) or ScP140 (*n* = 3) peptides. (**a**) Schematic schedule of injection protocols (5 times of 100 µg/injection/mice/day for 5 consecutive days; organs taken 3 days after the last injection). Blue arrows indicate the days of injections of P140 or ScP140 peptides and the red arrow indicates the day of sacrifice and collection of organs. (**b**) LysoTracker Green mean fluorescence intensity of CD19^+^/CD3^−^ B cells and CD19^−^/CD3^+^ T cells from the four study groups of mice as assessed by flow cytometry. (**c**) Expression levels of Transcription factor EB (TFEB) in purified B cells from the four study groups as examined by Western blotting. (**d**) Mean lysosomal pH values splenocytes of the four study groups of mice as measured using LysoSensor Yellow/Blue DND-160. The pH value was calculated using a standard curve shown in Supplementary Figure S1. (**e**) Measurement of proteolytic capacities of lysosomes from CD19^+^/CD3^−^ B cells and CD19^−^/CD3^+^ T cells of the four study groups of mice, as evaluated by flow cytometry in measuring Self-Quenched BODIPY Dye Conjugates of Bovine Serum Albumin (DQ-BSA)fluorescence intensity. In (**b**,**d**,**e**), each point represents the mean value of the experimental triplicates. In (**c**) each point represents one mouse, in total 7 CBA/J mice, 9 MRL/lpr mice, 6 MRL/lpr mice + P140 and 3 MRL/lpr mice + ScP140. One-way ANOVA Krustal-Wallis test was used to analyze the statistical significance. Error bars are ± SEM. ns means *p* values > 0.05.

Mice and treatment: MRL/lpr mice and CBA/J control mice were purchased from Charles River-France and Janvier Labs, respectively. MRL/lpr mice, 10–12 weeks old, received an intravenous administration of 100 μg P140 or ScP140 in 100 μL saline per mouse or 100 μL saline alone for 5 consecutive days. The organs (spleen or liver) were taken three days after the last injection (Figure 1a). Animal protocols were carried out with the approval of the local Institutional Animal Care and Use Committee (CREMEAS, Strasbourg, France) and the French Ministry of Superior Education, Research and Innovation (APAFiS/Project Authorization for the use of Animals for Scientific Purposes). According to our agreement, and taking into account the best European practices in the field, we took the necessary measures to avoid pain and minimize the distress and useless suffering of mice during the time of experiment and killing process. The ScP140 control peptide was not included in some of these experiments to limit the number of MRL/lpr that were used, according to the 3 Rs principle (Replacement, Reduction and Refinement) imposed by the European “Regulations and Ethical Considerations in Animal Experiments”. We recognize that, scientifically speaking, this is objectionable.

Antibodies and other reagents: The sources of antibodies used in this study were—LAMP2A (ab125068), LAMP1, GAPDH (ab9482), HSPA8 (ab51052), HSP90 (ab203126), Tomm20 (ab209951), all from Abcam (Cambridge, England), GBA (PA5-52319, ThermoFisher Scientific, Carlsbad, CA, USA), ATP6V0A1 (sc-374475, Santa Cruz Biotechnology, Santa Cruz, CA, USA), and TFEB (A303-673A-M, Bethyl, Montgomery, TX, USA). The antibody against total Tau (DA9) was a gift from Dr. P. Davies (The Feinstein Institute, Manhasset, NY, USA). Tau protein was prepared as described [34].

Determination of lysosomal properties: Mouse splenocytes were first incubated with 10 μg/mL DQ-Red BSA (ThermoFisher Scientific) for 2 h to allow the internalization of BSA within lysosomes, then washed with phosphate-buffered saline (pH 7.4), and stained with 100 nM LysoTracker Green DND-26 (ThermoFisher Scientific) for 15 min at 37 °C, before being surface-stained with CD19-phycoerythrin (PE) and CD3-PerCPCy5.5 antibodies (ThermoFisher Scientific) for 20 min at 4 °C. Fluorescence was measured with a BD FACSCalibur cytometer (BD Biosciences) and analysis was conducted with the FlowJo software (Tree Star, Ashland, OR, USA). B and T cells were selected as CD19^+^/CD3^−^ and CD19^−^/CD3^+^, respectively. The fluorescence intensity of DQTM Red BSA correlates with the degradation capacity of lysosomes [35], and the fluorescence intensity of LysoTracker Green indicates the volume of lysosomes [35]. The lysosomal pH of splenocytes was measured as previously described [22]. Some experiments (e.g., the measurement of TFEB levels) were performed with purified B cells obtained using a B cell isolation kit (Miltenyi Biotec, Bergisch Gladbach, Germany).

Enrichment and purification of lysosomes: Lysosome-enriched fractions were prepared from either the spleen or the liver of mice, as described previously (Section 3.1, step 1–5) [36]. They were either subjected to Western blotting or loaded at the bottom of a discontinuous metrizamide gradient for ultracentrifugation to obtain CMA^+^ and CMA^−^ lysosomes (Section 3.1, step 5–9). The integrity and recovery of lysosomes was determined using the hexosaminidase latency assay described earlier [36].

Uptake of Tau CMA substrate by intact lysosomes: Lysosomes isolated from mouse liver were incubated with purified Tau protein for 20 min at 37 °C in 3-(*N*-morpholino)propanesulfonic acid (MOPS) buffer (10 mM MOPS, pH 7.3, 0.3 M sucrose) [36]. At the end of the incubation, lysosomes were collected by centrifugation and washed, and the pellets were subjected to Western blotting. The uptake of Tau was estimated from three parameters defined as follows: “binding” was the amount of Tau measured in the absence of lysosomal protease inhibitors (Tau bound to the lysosomal membrane); “association” was the amount of Tau estimated in the presence of lysosomal protease inhibitors (Tau bound to the lysosomal surface + internalized Tau); “uptake” was calculated by subtracting “binding” from “association” [36].

Protein degradation in lysosomes: Lysosomes were incubated with a pool of [3H]-labeled cytosolic proteins in MOPS/dithiothreitol buffer (10 mM MOPS, pH 7.3, 0.3 M sucrose, 1 mM dithiothreitol, and 5.4 µM cysteine) in the presence of 0.1% (*v/v*) Triton to disrupt their membranes for 20 min at 37 °C. After precipitation in acid, proteolysis was calculated as the amount of acid precipitable radioactivity (protein) transformed to an acid-soluble state (amino acid) during the incubation [36].

Western blots: Total cell lysates were prepared in RIPA buffer (150 mM NaCl, 1% (*v/v*) nonyl phenoxypolyethoxylethanol-40, 0.5% (*v/v*) sodium deoxycholate, 0.1% (*v/v*) sodium dodecyl sulfate (SDS), 50 mM trishydroxymethylaminomethane (Tris), pH 8.0) containing protease and phosphatase inhibitors. Protein concentration was measured using BSA as a standard. Samples were subjected to SDS-PAGE, transferred to a nitrocellulose membrane, blocked with low-fat milk and incubated with primary antibodies overnight. The proteins were visualized by using peroxidase-conjugated secondary antibodies and chemiluminescent reagent (PerkinElmer) in an LAS-3000 Imaging System (Fujifilm). Densitometric quantification was performed on unsaturated images using ImageJ (National Institutes of Health). The values were normalized to the Ponceau staining, which is a method of choice when working with different cellular fractions as a single protein is most generally not equally distributed across fractions.

Statistical analyses: All numerical results are reported as the mean ± standard error of the mean (SEM). One-way ANOVA Krustal-Wallis test was used for analyzing the results between our groups that were of equal or different sizes and were compared two by two. Differences were considered to be statistically significant when *p* ≤ 0.05.

## 3. Results

### 3.1. Splenic Lysosomes Display Dysfunction in MRL/lpr Mice That Can Be Rescued in Part by P140 Treatment

A number of characteristics and properties of lysosomes were evaluated in the spleen of 10–12 week-old MRL/lpr lupus mice and CBA/J control mice of the same haplotype H2k that were treated or not with P140 peptide (Figure 1a). Compared to CBA/J mice, the labeling by LysoTracker Green, which accumulates in acidic compartments but is mostly independently of pH, was significantly increased in splenic CD19^+^/CD3^−^ B cells and to a lesser extent in CD19^−^/CD3^+^ T cells of MRL/lpr mice (Figure 1b), indicative of an apparent expansion of the acidic vesicular organelles compartment (size and/or number) in both cell subsets. This result is consistent with what we have previously observed [22]. Transcription factor EB (TFEB) expression levels were significantly increased, suggestive here of higher rates of lysosomal biogenesis [37,38] in the spleen of MRL/lpr mice (Figure 1c and Figure S1a). Using LysoSensor Yellow/Blue DND-160, a ratiometric probe specific for measuring lysosomal pH (independent of lysosomal volume), we observed a pronounced increase in the average lysosomal pH in MRL/lpr total spleen cells (Figure 1d) and B cells (Figure S1b,c). Treating mice with P140 according to the procedure described in Figure 1a significantly decreased the abnormally enlarged volume (size and/or number) of endo/lysosomal compartments and lysosomal biogenesis in B cells (Figure 1b). However, it had no effect on the lysosome size in T cells and was unable to significantly rescue the abnormal lysosomal pH in peptide-treated mice (Figure 1b,d).

Using DQ-red bovine serum albumin (BSA) as a cargo, we found that the endolysosomal activity of acidic organelles was globally disrupted in MRL/lpr with regard to normal mice (Figure 1e). In this assay, BSA that is labeled with BODIPY TR-X dye becomes fluorescent upon proteolysis in the lysosomes [38,39]. Compared to normal B and T cells, DQ-BSA fluorescence was significantly lower in MRL/lpr splenic B cells, while, in contrast, it was higher in T cells. P140 show no significant impact on these abnormalities (Figure 1e).

Taken together our experiments clearly pinpoint a number of defects (pH, volume, proteolytic capability) displayed by MRL/lpr splenic lysosomes with unquestionable consequences on protein cleavage by lysosomal proteases and antigen processing. In an impressive manner, some of these defaults could be rescued by treating sick MRL/lpr mice with P140 peptide even when only a very short period of five days’ treatment was applied, indicative of a very efficient in vivo protective effect of P140 on lysosomes.

### 3.2. Overexpression of LAMP2A in Splenic Lysosomes is Corrected by P140 Treatment

**Figure 2:**
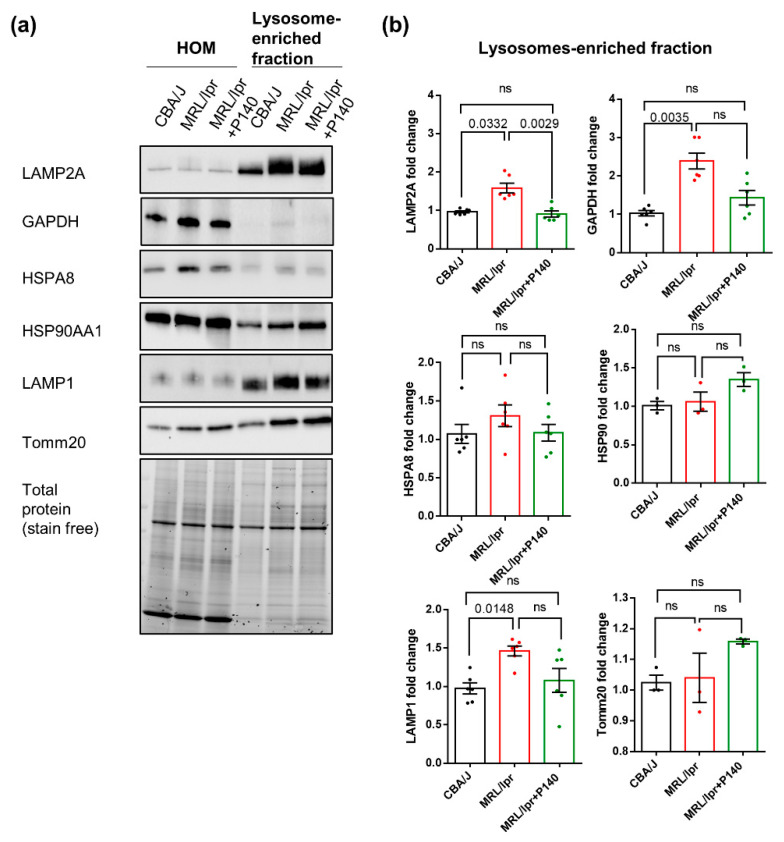
Expression of CMA markers in enriched lysosomal fractions from lupus mice and effects of P140 peptide: Cell homogenates (HOMs) and lysosome-enriched fractions were prepared from splenocytes of CBA/J, MRL/lpr and P140-treated MRL/lpr mice. (**a**,**b**) The protein expression levels of LAMP2A, GAPDH, HSPA8, HSP90, LAMP1 and Tomm20 were examined by Western blotting. Total protein normalization was used for quantification (stain-free method). *n* = 6 mice per group with triplicates for each sample. One-way ANOVA Krustal-Wallis test was used to analyze the statistical significance. Error bars are ± SEM. ns means *p* values > 0.05.

The expression levels of LAMP2A, HSPA8, HSP90AA1, effector proteins for CMA, and GAPDH, a well-validated CMA substrate protein, were measured by Western blotting to investigate the alterations of CMA in splenic lysosomal fractions of MRL/lpr mice and their potential rescue after P140 treatment. The translocase of outer mitochondrial membrane 20 homolog (Tomm20) was used as a mitochondrial marker and LAMP1 as a lysosomal marker that is not required for CMA. Note that, as recommended when working with isolated subcellular fractions and because of the expansion of the lysosomal compartments observed by the image-based analysis, all densitometric values for specific proteins in the different isolated fractions were normalized to total protein staining in the fraction rather that to a single lysosomal protein [40]. While no change of expression of any of these markers was detectable when total homogenate (HOM) fractions from healthy (CBA/J) and lupus (MRL/lpr) mice were analyzed (Figure 2 and Figure S2), a raised expression of LAMP1, LAMP2A and GAPDH was visualized in lupus lysosome-enriched fractions compared to control ones (Figure 2; 6 mice per group). The increased expression of LAMP1 (known to be regulated by TFEB) and LAMP2A (TFEB-independent regulation) is in agreement with the expansion of the endolysosomal compartment observed with LysoTracker, but in the case of CMA, higher levels of LAMP2A have been also shown to correlate with higher CMA activity [41]. Under normal conditions, only about 0.1% of total cellular GAPDH undergoes degradation via CMA and can be detected in lysosomes at a given time [42]. The higher amount of lysosomal GAPDH observed in the lupus mice group is also suggestive of its enhanced delivery to this compartment for degradation, and consequently of increased CMA activity. The expression levels of HSPA8 and HSP90 remained unchanged, as in the case of Tomm20 (supporting an equal loading of mitochondria in these experiments). Following P140 treatment, there was a trend toward a reduction in lysosomal levels of LAMP1 and GAPDH and a significant reduction in LAMP2A content whose levels were similar to those found in normal mice (Figure 2). These results strongly suggest that CMA in which LAMP2A represents a key element, could be modulated in vivo by the peptide.

### 3.3. P140 Decreases Direct Lysosome Uptake of CMA Substrates

In order to determine whether, in vivo, P140 affects CMA substrate translocation into lysosomes, next we engaged in a series of experiments using first a well-established system with freshly isolated lysosomes prepared from mice treated with P140 peptide or not [17,34]. This assay allows for the direct analysis of CMA independently of any other cellular proteolytic system. We analyzed the lysosomal uptake of the Tau protein as a model of the CMA substrate [43]. Since Tau is not normally expressed in the spleen, it is the ideal substrate to directly track a protein that is exogenously presented to lysosomes [43]. Lysosome fractions with high (+) or low (−) CMA activity were prepared by a series of differential centrifugation steps followed by flotation in discontinuous density gradients of metrizamide from the spleen of CBA/J healthy mice, MRL/lpr lupus prone mice and P140-treated MRL/lpr mice [16]. Unfortunately, and although there is a well-known enlargement of spleens in lupus-prone MRL/lpr mice [44], it was not possible to obtain enough CMA^+^ and CMA^−^ lysosomes separately, even by pooling 4–5 spleens, as attested by the low amount of lysosomes (measured by the expression of LAMP2A and glucocerebrosidase, GBA) recovered in the CMA^+^ fraction and unusually high levels of HSPA8 in the CMA^−^ fraction (Figure S3).

**Figure 3:**
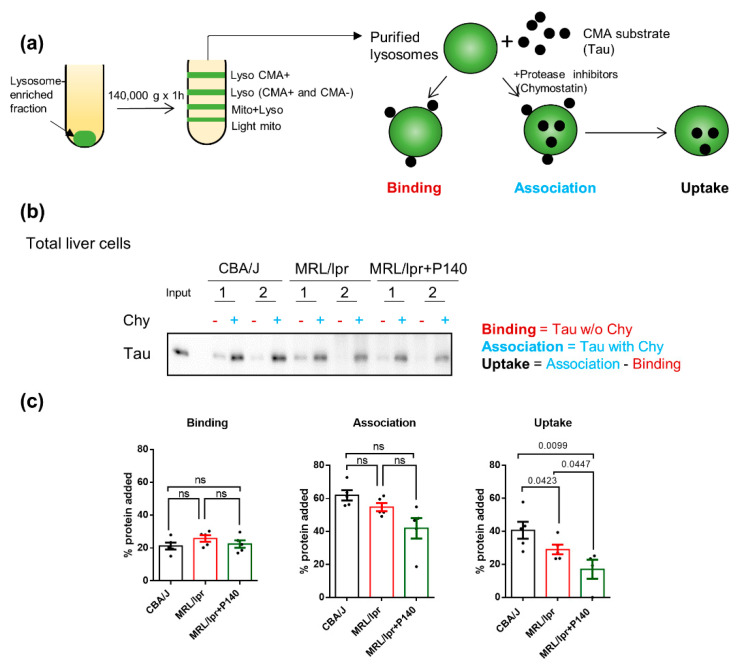
Effect of P140 peptide on the uptake of a prototypical chaperone-mediated autophagy (CMA) substrate into CMA-active lysosomal liver preparations. (**a**) Schematic presentation of purification of lysosomes and incubation assay to measure translocation of CMA uptake in vitro. Lysosome fractions with high (+) CMA activity were prepared from the liver of CBA/J, MRL/lpr and P140-treated MRL/lpr mice. Recombinant Tau protein (0.2 μg) was added for 20 min at 37 °C to lysosomes previously treated or not with the protease inhibitor chymostatin (chy; 100 μM for 10 min at 0 °C and diluted to a final concentration of 33 μM). Samples were subsequently treated for analysis by SDS-PAGE and Western blotting. (**b**) Western blots of the levels of Tau recovered from CMA^+^ lysosomes in the presence or not of chymostatin from the livers of CBA/J, MRL/lpr and P140-treated MRL/lpr mice. (**c**) Evaluation of the binding, association and uptake of Tau to CMA^+^ lysosomes recovered from the livers of the three mice groups, by using densitometry of the blots shown in (**b**). Five samples (each represented by one point) were examined for each study group. Each point represents a sample pooled from 2–3 livers. One-way ANOVA Krustal-Wallis test was used to analyze the statistical significance. Error bars are ± SEM. ns means *p* values > 0.05.

Although CMA can exhibit some tissue-specific functions, its general functions (activation upon stress and protein quality control) have been suggested to be conservative across different tissues and cell lines [45,46,47]. We therefore used instead lysosomes isolated from the liver—another organ with very low endogenous levels of Tau in CBA/J healthy mice, MRL/lpr prone mice and P140-treated MRL/lpr mice (Figure 3). Following the procedure depicted in Figure 3a, we measured lysosomal binding (in untreated lysosomes) and lysosomal internalization (in chymostatin-treated lysosomes) of the CMA substrate [34]. We did not observe differences in lysosomal binding of Tau among any of the three groups of mice—CBA/J, MRL/lpr or MRL/lpr—treated with P140 (Figure 3b,c). Analysis of Tau uptake revealed that CMA^+^ lysosomes from MRL/lpr mice livers were slightly less efficient in taking up Tau compared to CBA/J liver CMA^+^ lysosomes (*p* = 0.0423; Figure 3b,c). Interestingly, despite the lack of CMA hyperactivation in the lysosomes isolated from MRL/lpr mice, treatment with P140 resulted in a significant reduction in Tau uptake (*p* = 0.0447 vs. MRL/lpr and *p* = 0.0099 vs. normal CBA/J mice; Figure 3b).

**Figure 4:**
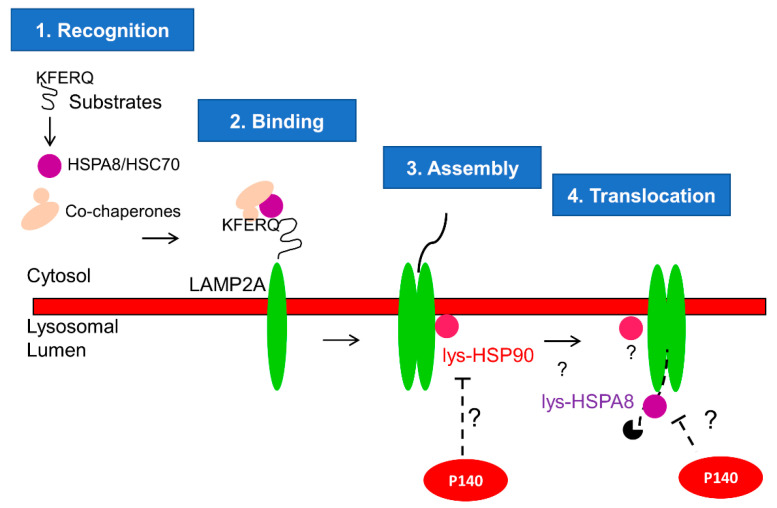
Proposed mechanism of P140 on CMA: CMA is a complex multistep process as described elsewhere in detail [17,18,36]. Here we focus on the CMA steps where P140 could play a role. Our results in this paper strongly suggest that P140 inhibits the substrate uptake step of CMA. The co-localization of P140 and HSPA8 was demonstrated earlier [24,27] by fluorescence and electron microscopy. It was not possible, however, to definitively determine that both co-localize into lysosomes or endosomes [27]. P140 interaction with HSPA8 was further demonstrated using surface plasmon resonance experiments and fishing experiments with living cells [23]. We place the effect of P140 in the lysosomal lumen because we have previously shown that incubation of intact lysosomes with P140 in vitro does not modify CMA activity [22]. These findings are in agreement with P140 being internalized by endocytosis and reaching the lysosomal lumen through endolysosome fusion. The co-localization of P140 and LAMP2A has been demonstrated using fluorescence microscopy experiments [24]. Again, due to the resolution of images, it was not possible to definitively determine that that both co-localize within lysosomes. However, the results obtained in this work with a highly purified fraction of lysosomes active for CMA provide strong support of this compartment being the target of P140. Therefore, we hypothesize that the lysosomal P140 encounters and inhibits lysosomal HSP90 and HSPA8, which are responsible of the assembly of LAMP2A multiplex and translocation of CMA substrate, respectively.

We have previously demonstrated that P140 inhibits CMA hyperactivity in MRL/lpr [3,22]. Here, we furthered our investigation and added an important step in our knowledge of mechanistic aspects of this inhibition by discovering that P140 hampers CMA at the substrate uptake step. CMA is a multistep process dependent on chaperone proteins HSPA8 and HSP90 that are located inside and outside of the lysosomal lumen [17,18,36]. We have previously reported that P140 is an inhibitor of HSPA8 and HSP90 in ex vivo and in vitro settings [24,27]. We therefore hypothesize that P140, which was found in lysosomes [22], could hamper the function of HSPA8 and/or HSP90 necessary for the assembly of LAMP2A multiplex and translocation of the CMA substrate (Figure 4).

The unexpected lower uptake activity of MRL/lpr CMA^+^ lysosomes compared to CBA/J ones could also be due, at least in part, to a particular fragility of lysosomes issued from MRL/lpr livers, an assumption we already made previously [3]. This hitherto hypothetic characteristic of MRL/lpr lysosomes was experimentally demonstrated here by showing that there was more hexosaminidase released in the media (Figure S4a) and less hexosaminidase activity inside the isolated lysosomes in the case of MRL/lpr lysosomes compared to CBA/J liver cells (Figure S4b), indicative of a higher number of broken lysosomes in MRL/lpr conditions. However, the percentage of fragile lysosomes is rather small (from 9% in CBA/J mice to 12% in MRL/lpr CMA-active lysosomes), which should not affect our further analysis of CMA substrate uptake in Figure 3. Lysosomal fragility was revealed during the step of their purification as we did not visualize it in the homogenates of MRL/lpr liver, where in contrast we found a significant increase in the total levels of hexosaminidase, in agreement with the observed expansion of the lysosomal system that we described before (Figure S4c). Lysosomal fragility in the MRL/lpr was not corrected by P140 treatment (Figure S4a,b), which makes the observed inhibitory effect of the peptide in Tau lysosomal uptake still valid since both groups of lysosomes were in similar conditions of membrane integrity.

In contrast with the deficiencies in the proteolysis of substrates internalized by endocytosis (BSA fluorescence) observed in MRL/lpr cells (Figure 1e), we found no difference in the global proteolytic activity of the CMA^+^ lysosomes, suggesting that the P140-induced reduction in lysosomal uptake was not a consequence of changes in the proteolysis extent of substrates once inside lysosomes but rather changes in their direct translocation across the lysosomal membrane (Figure S5). This observation is of particular importance considering the mode of action of P140. Effectively, if P140 does not downregulate binding or proteolysis, the uptake as shown in Figure 3c can then be claimed as the main CMA step affected by the peptide.

As shown in the lysosome-enriched fraction of MRL/lpr spleen, an immunoblot analysis of the CMA^+^ lysosomes from liver before incubation revealed that LAMP2A expression was significantly higher in MRL/lpr mice compared to CBA/J mice (*p* = 0.0022), supporting that in vivo CMA activity may be higher in their livers too. This increase in LAMP2A expression was reduced to a limited extent (however, not significantly) by treating MRL/lpr mice with P140 peptide. The same trend was observed in the case of HSPA8, while no effect could be visualized regarding GAPDH (Figure S6).

Another important result was the finding that in MRL/lpr livers; the balance between CMA^−^ and CMA^+^ lysosomes appeared oriented towards CMA-active lysosomes as they can be detected even as part of the CMA^−^ pool (Figure S6). Compared to CBA/J lysosomes, the content in HSPA8, the selective marker of CMA^+^ lysosomes, was higher in MRL/lpr CMA^−^ lysosomes than in the same fraction from CBA/J mice (*p* = 0.0232; Figure S6). The fact that GAPDH levels were also significantly higher in CMA^−^ lysosomes from MRL/lpr mice compared with CBA/J (*p* = 0.0432; Figure S6) suggests that part of the enhanced CMA activity observed in MRL/lpr could take place in the normal CMA^−^ lysosomes. In fact, a similar expansion of CMA^+^ lysosomes as expenses of the CMA^−^ group has been previously described in the early stages of aging [41]. Treatment with the P140 peptide resulted in a discrete reduction in HSPA8 in the CMA^−^ lysosomes from MRL/lpr mice that were no longer significantly different to the CBA/J lysosomes (Figure S6). This might indicate that in lupus conditions, CMA^−^ lysosomes retain more HSPA8 in their lumen to become more active in CMA, possibly to compensate for the overall higher lysosomal fragility. However, the effect of P140 peptide in reducing CMA activity seems to be more prominent at the level of substrate uptake than on the balance between the two lysosomal populations, further supporting the targeting of HSPA8 independently of the lysosomal subtype, since both types of lysosomes also receive endocytic material.

## 4. Discussion

In this work, we demonstrate for the first time that P140 inhibits CMA in vivo at the substrate lysosomal uptake step. This was shown by studying Tau uptake in a transport assay using lysosomes purified from the liver of MRL/lpr mice. As P140 did not downregulate proteolysis, we concluded that very likely this inhibition effect of the uptake is specific for CMA.

Our research has shown a consistent lysosomal malfunctioning in lupus splenocytes, manifested in the increase in endo/lysosomal volume, loss of acidification, reduced proteolytic activity against endocytosed substrates and TFEB expression. These lysosomal abnormalities have been simultaneously observed in aging, neurodegenerative diseases and other conditions where lysosomes are disordered or stressed [5,37,48]. However, our studies have revealed that the changes in the endo/lysosomal system in lupus-prone mice are much more complex than initially anticipated, likely due to the well-known heterogeneity of the various subsets of these compartments. Therefore, each of the different pathways that utilize these compartments (and their subsets), require separate attention. For example, we have identified a decrease in lysosomal degradation via DQ-BSA assay. However, when isolating the specific subset of lysosomes involved in CMA, we have found that their proteolytic activity is intact. In the former case, the DQ-BSA reaches mainly lysosomes that fuse with endosomes; while in the latter case, the subset of lysosomes might be different, likely being the population more involved in autophagy.

It was, however, perplexing that the ScP40 control could also decrease TFEB expression. We did not investigate this issue further in this study, because TFEB does not directly relate to CMA, our main focus in this work. The key components for CMA, such as LAMP2A and HSPA8, are not under TFEB regulation. Our working hypothesis is that the normalization of the upregulated levels of TFEB by P140 can exert a correcting impact on lysosomal function (LysoTracker intensity) and in the observed abnormal expansion of the endolysosomal system. The fact that ScP140 loses its beneficial effect on CMA even though it decreases TEFB level further supports that the observed TFEB changes, albeit interesting, are not the determinants of the P140-dependent changes in CMA described in this work. ScP140 is an “inactive” control of P140 in all the other experiments that we have performed so far [9,22,23,24,27,28]. Further studies are required to investigate the mechanism behind the observed changes in TFEB by the ScP140 control.

Due to the low quantity of spleen lysosomes we could isolate from lupus mice, a technical limitation we did not succeed to solve, we used liver cells for analyzing the uptake of CMA substrates. Liver cells were a good alternative to address the mechanistic effects of P140 on CMA^−^-related lysosomes in vivo. Firstly, CMA mechanisms are conserved across spleen, liver and other tissues, and the mechanism of action of P140 is through interaction with HSPA8, a step essential for CMA in all tissues. Secondly, as there are many subsets of lysosomes, using isolated liver lysosomes allows us to better focus on lysosome subsets normally involved in CMA rather than on the whole lysosomal population, a challenge that was impossible to reach when spleens were used as source of lysosomes.

The isolation of lysosomal subpopulations from liver has also enabled us to identify an increase in the percentage of CMA-active lysosomes (containing HSPA8), suggesting that an increase in CMA activity per lysosome along with a higher number of lysosomes recruited to this autophagic pathway are responsible for the overall higher CMA activity in lupus. It is possible that the previously described reduction in macroautophagy with the subsequent lower number of lysosomes fusing to autophagosomes contribute to this higher number of CMA-active lysosomes [49]. Interestingly, although the isolation of lysosomes from spleen yielded a very low recovery unsuitable for to carry out the uptake experiments we planned, immunoblots of those fractions also demonstrated an increase in HSPA8 and LAMP2A in the CMA^−^ lysosomes from MRL/lpr spleens (Figure 3) suggesting that the imbalance toward CMA^+^ lysosomes may be a systemic feature in lupus. The swift of lysosomes toward a different pathway is actually not unusual and it is utilized by cells to adapt to the autophagic demands of the changing environment. Thus, for example, under basal conditions the fraction of CMA^−^ active lysosomes in liver is about 30% of all lysosomes, while after two days of starvation it can reach up to 80%. This increase in CMA^−^-active lysosomes does not require lysosomal biogenesis but is rather a result of acquisition of HSPA8 by otherwise CMA^−^-inactive lysosomes.

In contrast to spleen, where the levels of both CMA components and CMA substrates in lysosomes were higher in lupus-prone mice and the P140 peptide reduced their levels, in the case of the liver, we were not able to detect increased CMA activity in isolated lysosomes. However, lysosomes from MRL/lpr livers had higher LAMP2A and HSPA8 levels of expression. It is likely that the higher fragility that we observed in MRL/lpr lysosomes partially compromises their CMA activity upon isolation. Interestingly, even under these conditions, P140 was still able to reduce the uptake of CMA substrates into lysosomes. One other plausible explanation for the differences between the in vivo situation and isolate lysosomes could be that only the CMA of specific substrates is accelerated in the lupus context and that substrates such as Tau or GAPDH are not among those. A future line of investigation will be therefore to identify CMA substrates that are closely related to the lupus pathophysiology to reanalyze this central issue.

## 5. Conclusions

A number of activators and inhibitors of autophagy have been described [5,50,51,52,53]. In almost all cases, their fine specificity still remains to be determined. Few of them interact with one single target of a specific autophagic process (e.g., macroautophagy, CMA, mitophagy, others). This certainly explains the current limited therapeutic scope of molecules that influence deregulated autophagy processes. However, recent progress led us to envisage that strategies based on autophagy as a therapeutic target might have potent applications for specific interventions in the near future. P140 peptide that seems to modulate excessive CMA selectively is among the tools that show many promises in this regard. Despite HSPA8, the main target of P140, being involved in many other proteostasis pathways, the selectivity of P140 is conferred in this case by its internalization by endocytosis, which makes it accessible only to the HSPA8 located in the lysosomal lumen, which to date has only shown to be necessary for CMA. Our studies have highlighted its safety and its effectiveness in reducing clinical and biological features in mice and patients with lupus without exerting a harmful global immunosuppressive effect on immune defenses [25,26]. P140 is also a potent pharmacological molecule in murine models that mimics other autoinflammatory diseases, such as Sjögren’s syndrome and neurologic autoimmune disorders [9,54,55]. Demonstrating that P140 effectively interferes with CMA in vivo was therefore decisive to reinforce its therapeutic potential.

## Supplementary Materials

The following are available online at https://www.mdpi.com/2073-4409/9/10/2328/s1, Figure S1: Measurement lysosomal function in B cells of control and MRL/lpr mice., Figure S2: Expression of CMA markers in spleen cell homogenates from lupus mice, and effects of P140 peptide., Figure S3: Expression of CMA markers in spleen cell homogenates and purified lysosomes from spleens of lupus mice, and effects of P140 peptide., Figure S4: Evaluation of the integrity and recovery of lysosomes in lupus mice after purification. Figure S5: Lysosomal matrix proteolysis of a pool of cytosolic proteins in lupus mice, and effect of P140. Figure S6: Expression of CMA markers in the homogenates and purified lysosomal fractions from lupus mice, and effects of P140 peptide.
