# Supplementary material for: In Vivo Remodeling of Altered Autophagy-Lysosomal Pathway by a Phosphopeptide in Lupus
Source: Cells. 2020 Oct 20;9(10):2328. doi: 10.3390/cells9102328 (PMC7589999; doi:10.3390/cells9102328)
Supplement: Supplementary file 1 [file cells-09-02328-s001.pdf]

cells

MDPI

Supplementary Material

In vivo remodeling of altered autophagy-lysosomal pathway by a phosphopeptide in lupus

Fengjuan Wang, Inmaculada Tasset, Ana Maria Cuervo, and Sylviane Muller

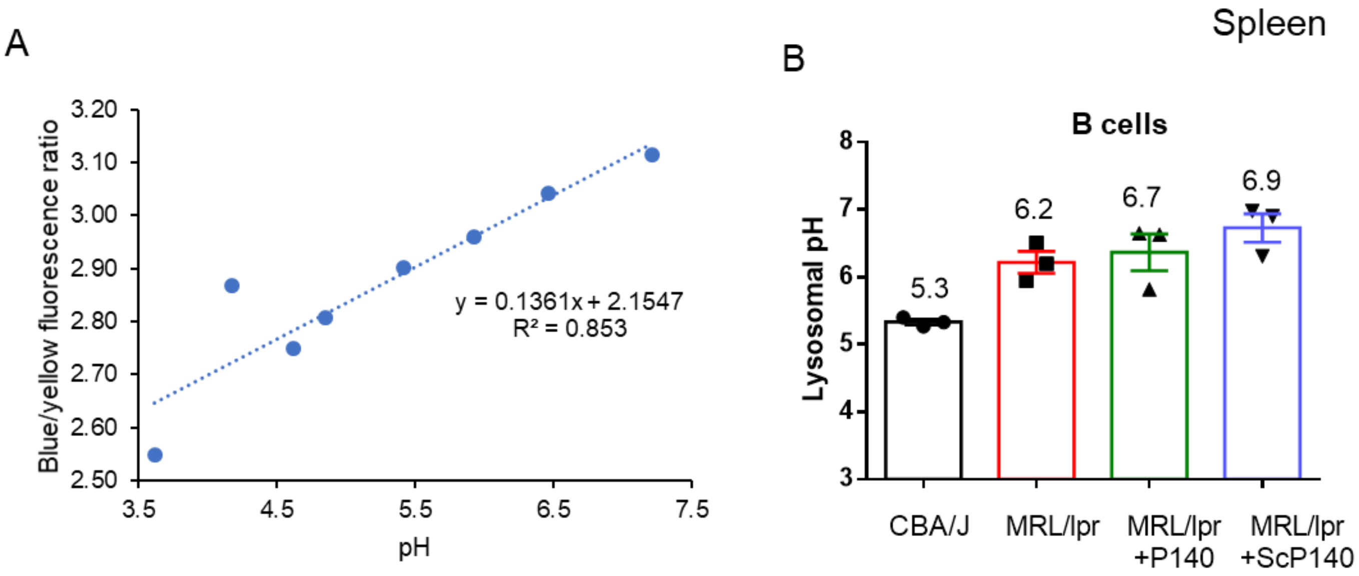

Supplemental Figure S1: Measurement lysosomal function in B cells of control and MRL/lpr mice. (A) Standard curve corresponding to the ratio of blue (460 nm) and yellow (535 nm) fluorescence of LysoSensor Yellow/Blue staining *versus* pH values. (B) Lysosomal pH of B cells purified from CBA/J, MRL/lpr, and peptide-treated MRL/lpr. Three mice were used per group and each point represents the average value of 6 experimental replicates. Error bars are ± SEM.

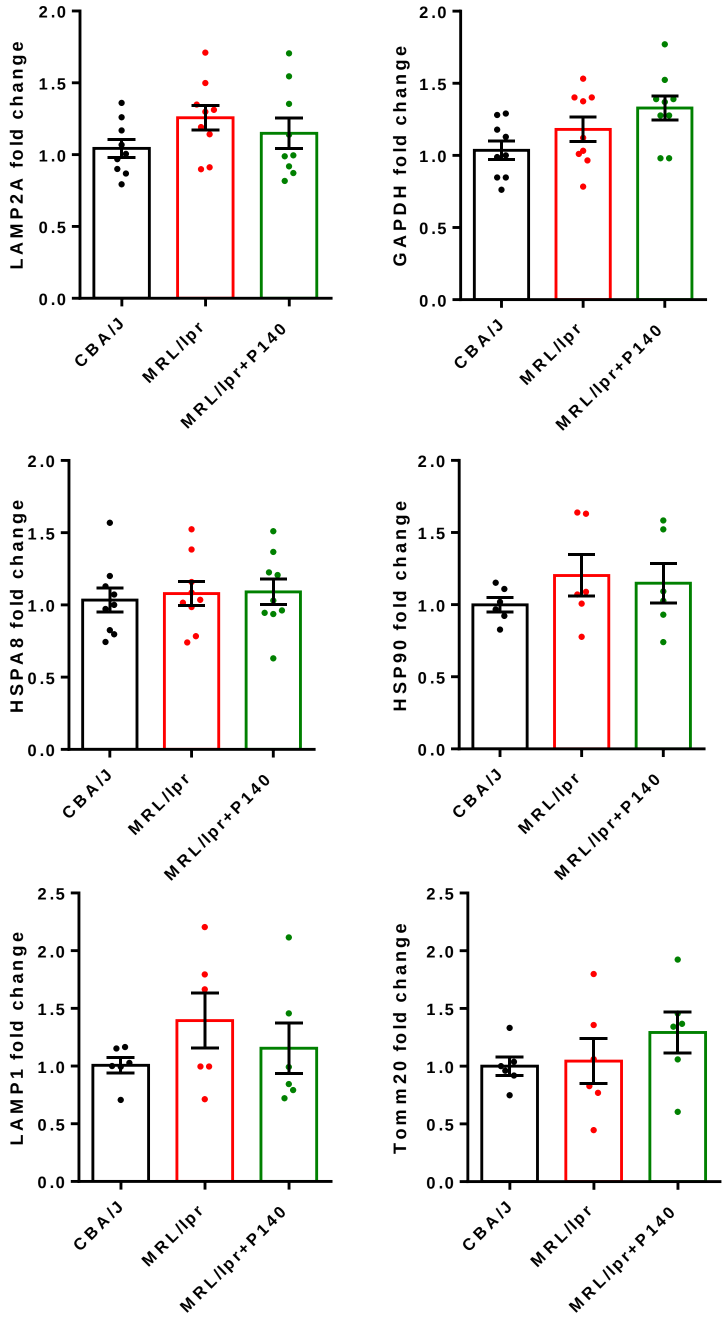

Supplemental Figure S2: Expression of CMA markers in spleen cell homogenates from lupus mice, and effects of P140 peptide. The protein expression levels of LAMP2A, GAPDH, HSPA8, HSP90, LAMP1 and Tomm20 in the homogenates from CBA/J, MRL/lpr and P140-treated MRL/lpr (MRL/lpr+P140) were examined by western blot and quantified as in Figure 2.

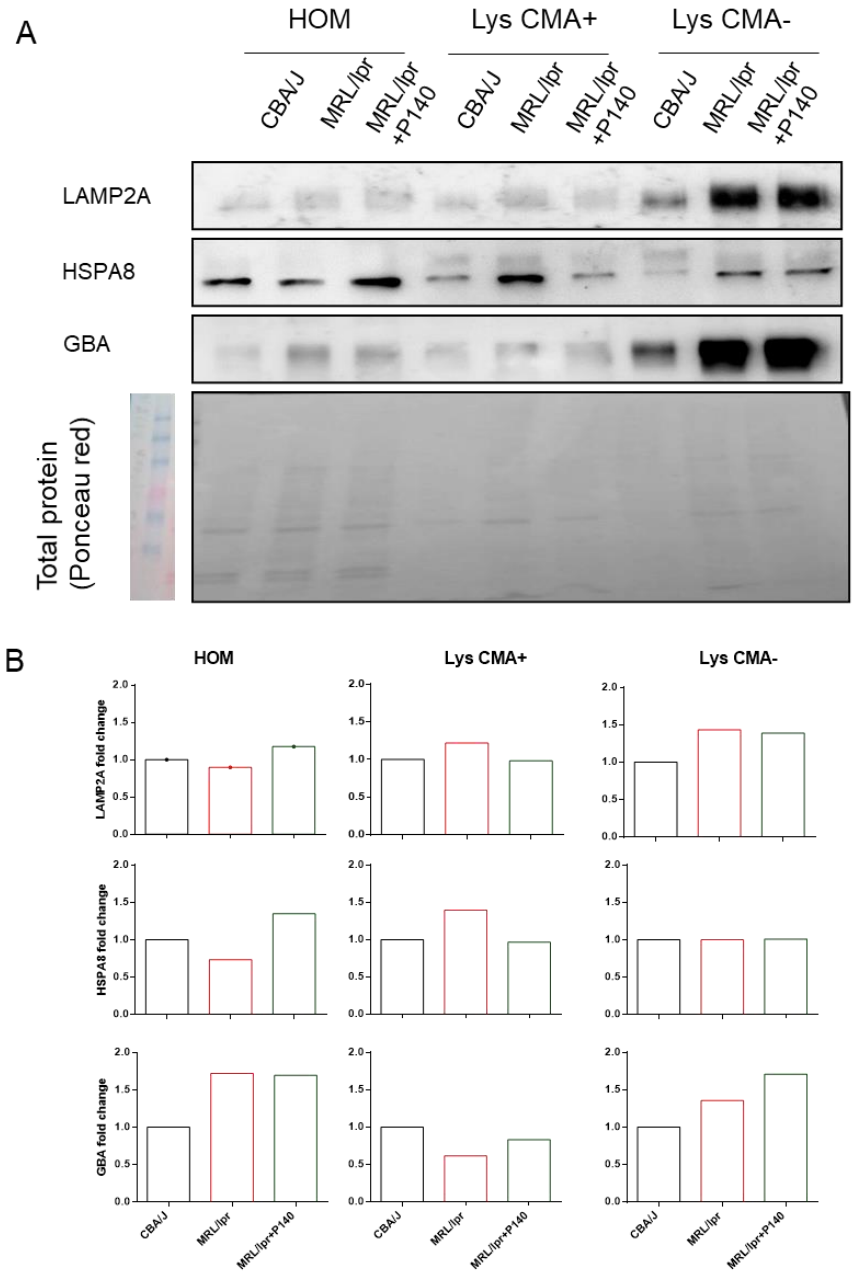

Supplemental Figure S3: Expression of CMA markers in spleen cell homogenates and purified lysosomes from spleens of lupus mice, and effects of P140 peptide. (A) Western blot of of LAMP2A, HSPA8 and GBA from cell homogenates, CMA active and CMA inactive lysosomes generated from the spleens of CBA/J, MRL/lpr and P140-treated MRL/lpr mice. The purification of CMA+ and CMA- lysosomes from the spleen was done as described in Figure 3A for liver. The total protein content was evaluated by Ponceau red staining. (B) Quantification of Western blots shown in (A) using densitometry (Image J software). The lysosomal marker GBA and CMA makers LAMP2A and HSPA8 were revealed using specific antibodies.

Abbreviations: GBA, glucocerebrosidase; HOM, cell homogenates; Lys CMA+, CMA active lysosomes; Lys-CMA-, CMA inactive lysosomes.

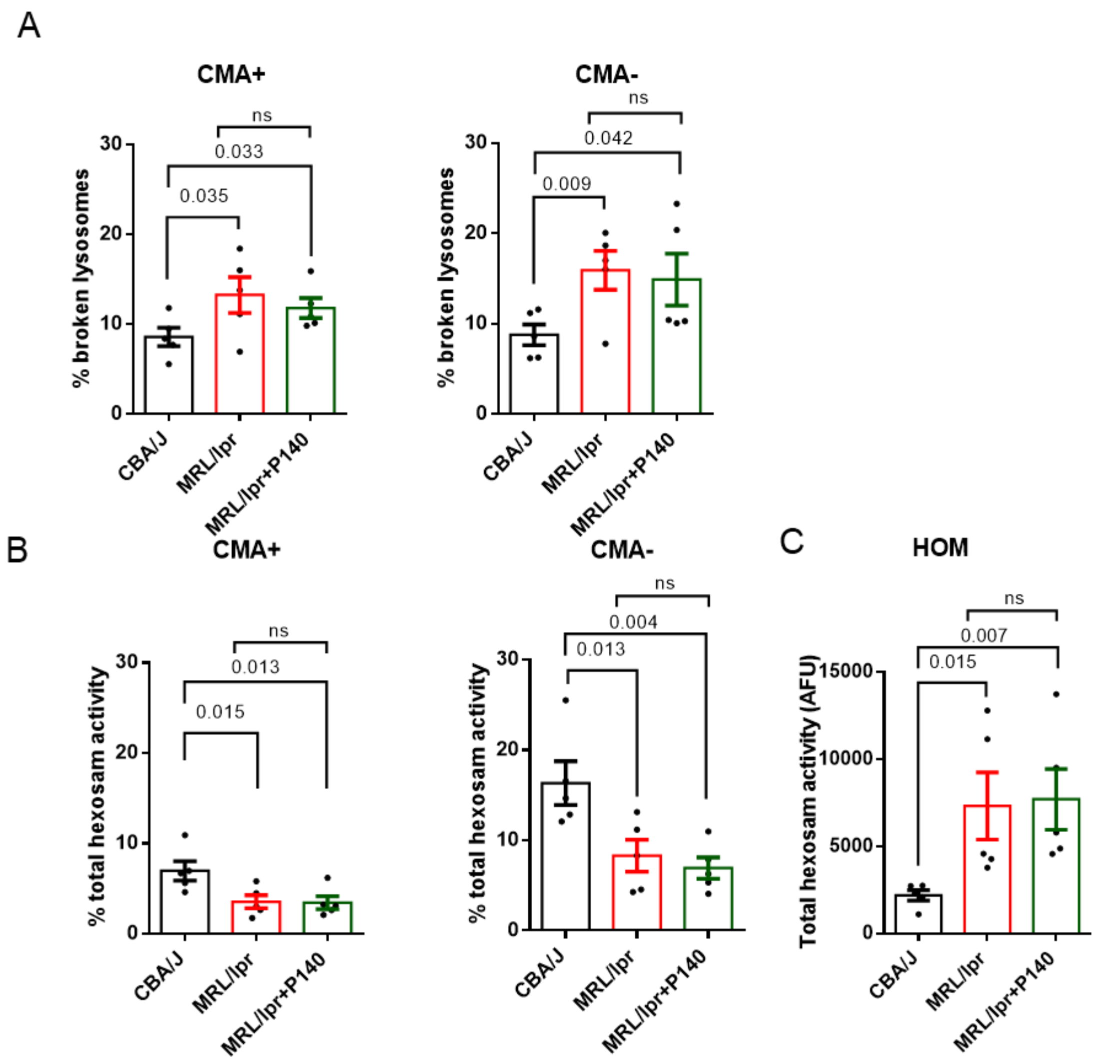

Supplemental Figure S4: Evaluation of the integrity and recovery of lysosomes in lupus mice after purification. CMA+ and CMA- lysosomes isolated from the livers from the three study groups of mice were recovered after centrifugation, and hexosaminidase activity was measured after centrifugation to sediment the lysosomes in the pellets, supernatant and total (pellet + supernatant) homogenate fractions. (A) The levels of broken lysosomes were calculated as the percentage of hexosaminidase activity detected in the supernatant over those of the total. (B) Recovery of lysosomes was calculated as the percentage of hexosaminidase activity detected in lysosomes over that in the corresponding homogenates. (C) The total hexosaminidase activity from cell homogenate is plotted in arbitrary fluorescence units. Each point represents one sample after pooling 2-3 livers together; 5 samples were examined for each group. Non-parametric two-way ANOVA was used to analyze the statistical significance. Error bars are ± SEM. ns means P values >0.05.

Abbreviations: AFU, arbitrary fluorescence units; CMA+, CMA active lysosomes; CMA-, CMA inactive lysosomes; hexosam, hexosaminidase; HOM, homogenates

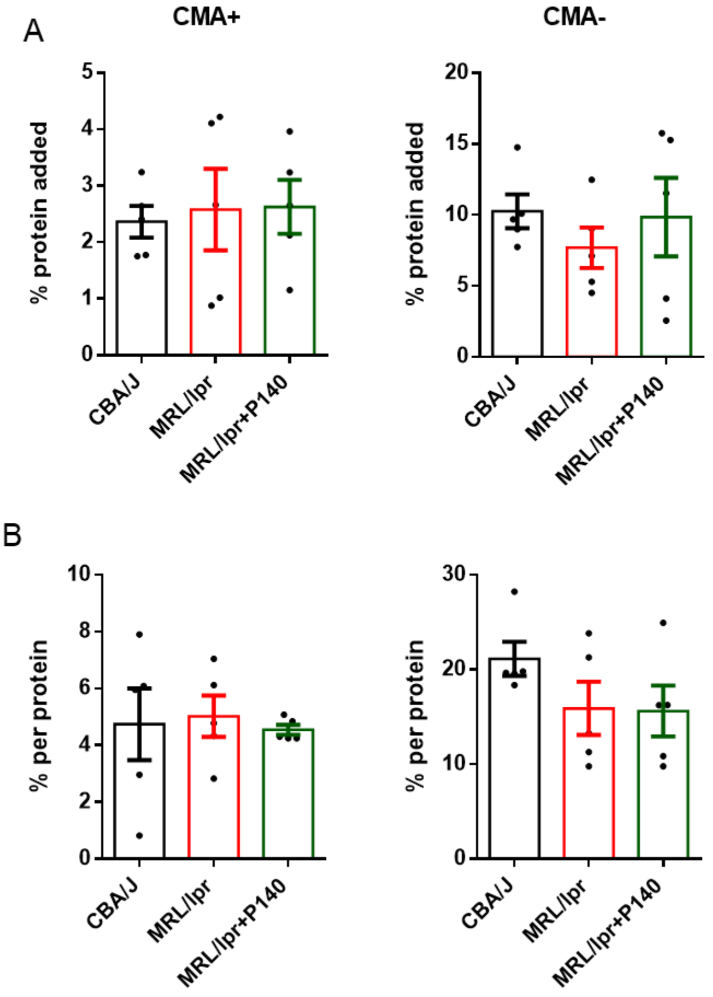

Supplemental Figure S5: Lysosomal matrix proteolysis of a pool of cytosolic proteins in lupus mice, and effect of P140. Lysosomes with high or low CMA activity were isolated from the livers of CBA/J, MLR/lpr mice and P140-treated mice treated, incubated with detergent to disrupt their membranes and allow free access of proteases to substrates, and incubated with a pool of tritiated cytosolic proteins for 20 min at 37˚C. At the end of the incubation, samples were precipitated with trichloroacetic acid and filtered to evaluate proteolysis as the percentage of acid soluble radioactivity (protein) converted to acid soluble activity (amino acids) at the end of the incubation. The absence of changes in the proteolytic activity of CMA+ lysosomes was measured after completely disrupting their membrane (to make substrates directly accessible to the proteases) and incubating with a pool of [^{3}H]-labeled cytosolic proteins. Proteolysis extent was calculated as the fraction of radioactivity present in proteins converted into radioactivity in soluble amino acid residues, resulting from protein breakdown, at the end of the incubation. Values in (A) are corrected per volume of lysosomes added and in (B) per 10 mg lysosomal protein added. Each point represents one sample after pooling 2-3 livers; 5 samples were examined for each group; 3 replicates were performed for each sample. Non-parametric two-way ANOVA was used to analyze the statistical significance. Error bars are ± SEM.

Abbreviations: CMA+, CMA active lysosomes; CMA-, CMA inactive lysosomes.

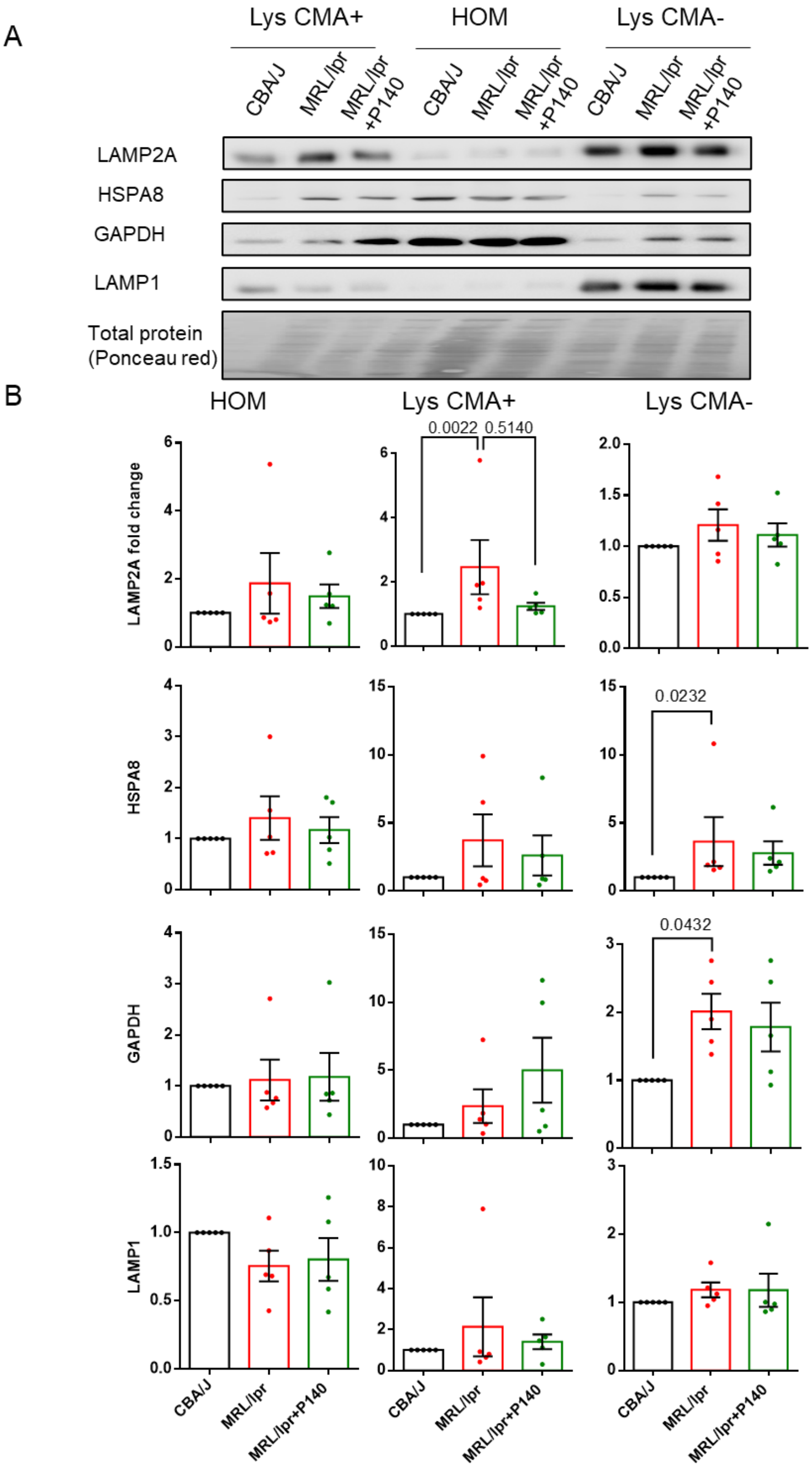

A
Lys CMA+
HOM
Lys CMA-
CBA/J
MRL/lpr
MRL/lpr +P140
LAMP2A
HSPA8
GAPDH
LAMP1
Total protein (Ponceau red)
B
HOM
Lys CMA+
Lys CMA-
LAMP2A fold change
0.0022
0.5140
HSPA8
0.0232
GAPDH
0.0432
LAMP1
CBA/J
MRL/lpr
MRL/lpr+P140

Supplemental Figure S6: Expression of CMA markers in the homogenates and purified lysosomal fractions from lupus mice, and effects of P140 peptide. (A) Western blots of LAMP2A, GAPDH, HSPA8 and LAMP1 from cell homogenates and CMA active and inactive lysosomes purified from the livers of CBA/J, MRL/lpr and P140-treated MRL/lpr. Ponceau red staining was used to reveal the total protein content. (B) Quantification of (A) using densitometry (Image J software, National Institutes of Health). Each point represents one sample after pooling 2-3 livers; 5 samples were examined for each group. Non-parametric two-way ANOVA was used to analyze the statistical significances. Error bars are ± SEM. P values are indicated for the statistically significant differences only.

Abbreviations: Lys CMA+, CMA active lysosomes; Lys CMA-, CMA inactive lysosomes; HOM, homogenates.
